# Positional Goods and Social Equality: Examining the Convergence Thesis

**DOI:** 10.1007/s11158-023-09581-8

**Published:** 2023-05-03

**Authors:** Devon Cass

**Affiliations:** Institute of Philosophy, Nova University of Lisbon, Lisbon, Portugal

**Keywords:** Positional goods, Social equality, Relational equality, Priority, Sufficiency, Levelling down

## Abstract

Several philosophers argue for the ‘convergence thesis’ for positional goods: prioritarians, sufficientarians, and egalitarians may converge on favouring an equal (or not too unequal) distribution of goods that have positional aspects. I discuss some problems for this thesis when applied to two key goods for which it has been proposed: education and wealth. I show, however, that there is a variant of the thesis that avoids these problems. This version of the thesis is significant, I demonstrate, because it applies to a person’s status as a citizen, which I suggest is the central concern of social or ‘relational’ egalitarianism.

## Introduction

A range of goods—*e.g.,* education, wealth, legal representation, political influence—are often regarded as ‘positional’. What these goods have in common is that their value to their possessor depends, at least in part, on how much of them they have compared to others, or where they are positioned in the distribution. While the notion was originally introduced in economics, several philosophers argue that the nature of positional goods bears important implications for the theory of justice; in particular, for the choice of distributive principle: *i.e*., deciding between equality, priority, sufficiency, and so on.^1^ The suggestion is that the positional aspects of some goods supplies a new line of support for their equal, or not too unequal, distribution and a rejoinder to some of the critiques that favour principles of priority or sufficiency over equality.^2^ These non-egalitarian views (priority and sufficiency), so it is argued, have reason to support removing or reducing inequalities of goods with positional aspects, on the grounds that doing so may be the best or only way to benefit those who are badly off in absolute terms, or give all ‘enough’ by the relevant criterion.^3^ Call this the *convergence thesis*.

In this paper, I examine this thesis and take up some concerns that suggest it may fail to hold in a range of cases. I argue that despite these challenges, the convergence thesis is particularly compelling when applied to a case not previously considered: the good of status, and in particular people’s status as citizens, which I suggest is the central concern of social or ‘relational’ egalitarianism. The account I develop brings to light an important distinction between two different kinds of positional goods, or two versions of the idea that positional goods are such that their value to an agent ‘depends on relative position’. For most goods commonly regarded as positional, there is only a contingent connection between relative position and the good’s value to an agent. Greater education than others often provides an advantage on the job market—but only because of contingent factors such as the nature of existing hiring practices, as I will discuss in what follows. By contrast, other goods are positional in a stronger sense. These goods are *defined by*, or *consist in,* occupying a particular relative position. For example, the good of placing first in a race is defined by ranking above everyone else. Along these lines, I develop a distinction between what I call ‘position-sensitive’ and ‘position-defined’ goods. I show that that the case for the convergence thesis is more compelling for goods of the second kind. This claim is significant, I argue, because a person’s status as a citizen—their ‘civic status’—is a position-defined good in this sense. Moreover, since a range of other goods are *status-conferring—i.e.*, they contribute to a person’s civic status, these other goods have position-defined aspects as well.

The paper is in three parts. In part one, I discuss some existing arguments for the claim that goods such as education and wealth have positional aspects and that, as a result, prioritarians and sufficientarians have reason to support their equal, or not too unequal, distribution. In part two, I discuss how the significance of these arguments may be limited as a result of their position-sensitive nature. For position-sensitive goods, it may be that benefits to the worse off can be achieved in two ways other than equal distribution: by growing the supply of the good (or the supply of what it is used for obtaining) or mitigating or removing its positional aspects. Since these strategies may also benefit the worse off, they may be favoured by prioritarians or sufficientarians, and so the convergence thesis will fail to hold in a range of cases. In part three, I propose an account of position-defined goods, and show that for goods of this kind, neither growth nor removal of their positional aspects is a possible strategy. I then characterize an important position-defined good: status—and in particular, ‘civic status’—and I discuss its significance for the convergence thesis.

## The Convergence Thesis: Education and Wealth

Harry Brighouse and Adam Swift focus their argument for the convergence thesis primarily on education.^4^ Education is a positional good, they point out, because one’s chances on the job market are largely determined by how educated one is relative to others. A bachelor’s degree will serve as a better means to employment if others are less qualified, worse when others are more qualified, other things being equal. To be sure, there are other valuable aspects of education that are not positional, such as the intrinsic value of learning a subject, which one could enjoy to a greater or lesser extent in isolation. More precisely, then, we can say education-as-a-means-to-employment is a positional good: its value to an agent depends on where they are positioned in the distribution.^5^

Next consider wealth. Philosophers at least since Adam Smith have noted that possessing a relatively low amount of wealth or material goods may cause damage to a person’s self-respect and a loss of inclusion in social life.^6^ A particular absolute level of wealth may provide a person adequate conditions for self-respect and social inclusion in a context where others do not possess a great deal more. But in another context with large differences of wealth, some may suffer stigmatization, living with the common awareness that they are judged as ‘less-than’ or inferior to others.^7^ This might threaten their self-respect understood, as Rawls suggests, as involving ‘a person’s sense of [their] own value, [their] secure conviction that… [their] plan of life…is worth carrying out’ and ‘a confidence in one’s ability…to fulfill one’s intentions’.^8^ Further, these effects of relative poverty may undermine social inclusion, understood, as Brighouse and Swift suggest, as ‘people’s ability to participate in the communal life and shared practices of their society’.^9^

The suggestion, then, is that wealth has positional aspects as a means to self-respect and social inclusion. To be sure, there is another sense in which wealth, as a means to *resources,* is not positional, since some resources (*e.g.,* food and shelter) have value in absolute terms, regardless of what others have. Bearing this in mind, it is again more precise to say that wealth has positional aspects in the ways identified, or that wealth-as-a-means-to-self-respect, or wealth-as-a-means-to-social-inclusion, is a positional good.

It is worth noting how the concern with self-respect and social inclusion may come apart. While it might be empirically likely that social exclusion will damage people’s self-respect, it is not necessary that this effect is realized for social exclusion to be found objectionable. Even if relative poverty is not ‘felt’ by people, in the sense that they do not suffer damage to their self-respect, we may nonetheless find a loss of social inclusion problematic. Consider, for example, that social exclusion may involve a denial of what T. M. Scanlon calls ‘associational goods’, or opportunities for valuable association with others: along with being marked out as inferior, the relatively poor may be seen as ‘less eligible to be co-workers, potential friends, possible marriage partners, or even neighbors’.^10^ A related aspect of social exclusion involves a loss of *agency*. Being regarded as ‘credible’, or as ‘somebody’ worthy of respect and taken seriously, often requires that people are able to present themselves in ways that require a particular amount of wealth. In the contexts discussed by Smith, this involved wearing a linen shirt or leather shoes. In our context, possession of a Smartphone (or at least access to the Internet), a credit card, and an address—all are all plausibly needed to be a ‘normal functioning agent.’^11^ These agency-impairing effects of relative poverty provide an explanation for why some people ‘waste’ money on things like iPhones and designer clothing. Such items can make a real difference for whether one is able to appear as ‘someone’ and be taken seriously.^12^ Importantly, these exclusionary effects are not a result of people being poor in absolute terms (no one needs an iPhone to survive). Rather, the thought is that as societies become wealthier and inequality widens, the bar for appearing as ‘someone’ may become higher. We could for instance imagine a variation of Smith’s example: a super-wealthy society, where social attitudes are such that people need *cashmere* shirts and *crocodile leather* shoes to appear as ‘someone’ and function as a ‘normal’ member. The concern, then, is that relative differences of wealth alone may cause a loss of social inclusion, which may also (or may not) harm people’s self-respect. What it means for a person to have ‘enough’ wealth cannot be specified in absolute terms independently of what others have, but instead depends on the difference between their level of wealth and that of others.

These observations about education and wealth are taken to support the convergence thesis: one does not need to be an egalitarian to support removing or reducing inequalities of goods with positional aspects.^13^ Since education and wealth have positional aspects in the ways described, concern for the worse off, or giving all enough, may support removing or at least reducing inequality with regard to these goods. Allow one group in society a much higher level of education, and the job prospects of the rest will suffer. Allow one group in a society great wealth, and the relatively poor may suffer harm to their self-respect and a loss of social inclusion. With regard to these positional goods, then, if we want to benefit the worse off, or give all enough, it may not be adequate to simply give them more of the good in question. What may be required is that the distance between them and others is not too great. On this basis, an equal, or not too unequal, distribution of these goods may be supported by prioritarians and sufficientarians. In some cases, priority and sufficiency may even support removing advantages from the better off, even when they cannot be transferred to others. As Scanlon notes, ‘the aim of avoiding stigmatization can in principle provide a reason for eliminating the benefits of the better off (or for wishing that they had never been created) even if these cannot be transferred to the worst-off’.^14^ Similarly, the aim of improving the job prospects of the worse off, or giving them ‘enough’ of an opportunity to compete with others, may support eliminating benefits enjoyed by an educated elite, even when these benefits cannot be transferred to others.

## Growth and Depositionalization of Position-Sensitive Goods

The convergence thesis is significant because it suggests that with regard to positional goods there may be less at stake than is commonly thought between seemingly opposed principles of justice. Equal, or not too unequal, distribution of positional goods may be supported on the widely accepted basis of concern for the worse off. However, the convergence thesis may be significantly limited, at least for goods such as education and wealth. This is because these goods are only ‘position-sensitive’. There are two features relevant for our purposes that characterize position-sensitive goods. The first is that the supply of the good (or what it is used for obtaining) can be grown; the second is that there is only a contingent connection between relative position and the good’s value, and so this connection can be weakened or eliminated entirely. Education and wealth share these features, as we will see, and this means that the convergence thesis may fail to hold in a range of cases.

### Education as a Means to Employment

In the case of education-as-a-means-to-employment, greater equality may be one way to benefit the worse off. But is it the only way? Not necessarily, as Christopher Freiman argues, if permitting inequality creates growth and increases the social product.^15^ The worse off may be benefited by growing the relevant end-use good of education: employment.^16^ Insofar as permitting inequalities of education increases the quantity and quality of jobs available to the worse off, the convergence thesis no longer holds. Priority for those worse off, or giving each enough, may in such cases be better served by allowing instead of removing inequality.^17^

To be sure, however, it seems unlikely that any degree of inequality will benefit the worse off, or that excessive advantages of a privileged elite will necessarily ‘trickle down’ to society’s poorest members. So, removing advantages from those at the top may still be the best way to improve the situation of the worse off or give all enough in some cases. But on the other hand, it is not implausible that *some* inequalities of education will allow for the creation of new industries, which will lead to the creation of more and better employment opportunities for the worse off.^18^ In cases where this possibility holds, the convergence thesis does not, since prioritarians and sufficientarians might favour an unequal distribution that provides even greater benefits for the worse off, and greater aggregate benefits, than an equal distribution. It may be, too, that *egalitarians* would favour growth-promoting inequality of particular goods such as education, if this brings about equality of wellbeing, or whichever good is taken to ultimately matter for justice.

In response, it might be argued that at any given point in time, the number of jobs available is fixed, so growth is not a salient strategy. While this makes sense, it may be replied that we ought to be more interested in principles of justice for political institutions persisting through time, and not only for fixed points or ‘time-slices’. Nevertheless, as a matter of non-ideal theory, the fixed supply of employment opportunities may be a durable feature of our societies for the foreseeable future. For that reason, then, the possibility of growth may not entirely undermine the significance of the convergence thesis.

While the convergence thesis may nonetheless hold in a range of cases, then, this range will also be limited to a certain extent by the possibility of growth. This exact range, however, cannot be determined by political philosophy alone, depending as it does on empirical facts. While the significance of the convergence thesis could thus be evaluated by engaging with the relevant empirics, I will not pursue that issue here.

In addition, there is a second way the convergence thesis may fail to hold, which involves what we might call ‘depositionalization’. It is not a necessary truth that having a better education relative to others gives one better prospects on the job market. Because of this, it may be possible to remove or mitigate education’s positional aspects. As Brighouse and Swift note,

it is not simply having more education that makes the person’s income prospects better. It is having more education in an environment in which that causal link holds. We could eliminate the causal link between relative education and absolute income by equalizing wage rates. We could reduce the causal link between relative education and absolute chances of getting interesting and responsible jobs by reducing the stigma attached to nepotism, by allocating jobs by lottery, or by reforming the job structure to make jobs more equally interesting and responsible.^19^

While total wage equalization, nepotism, or the assignment of jobs through lottery are not likely to find much support in liberal democracies, they point towards a relevant possibility. There may be more desirable forms of depositionalizing education. For example, it may be possible to implement policies that require employers to take into consideration only educational qualifications that are necessary for the job, and to avoid assigning positive evaluation to those in excess.^20^ We also might seek to implement policies or to encourage social practices that allow for other kinds of credentials to be evaluated positively. Along these lines Debra Satz notes that in places such as Sweden, it is possible ‘to rise to high political positions because of one’s experiences in the labor union movement’, for example, whereas in other places these positions may tend to be almost exclusively filled by those with elite educations.^21^

Policies that aim to implement these forms of depositionalization may be favoured over equal distribution for a number of reasons, especially if the latter were to require removing advantages from the better off without transferring them to others. For one, removing educational advantages would remove the non-positional benefits of allowing high levels of education in society such as the availability of expertise. Faced with a choice of removing the educational advantages of the better off versus mitigating the extent to which those advantages make labour market competition unfair for the worse off, prioritarians and sufficientarians would likely favour the latter. And again, it is also possible that egalitarians could prefer depositionalizing rather than equalizing particular goods such as education if doing so were a better way to bring about equality of wellbeing or whatever good is ultimately taken to matter. So, the convergence thesis may also fail over a range of cases in which depositionalization is possible.^22^

Again, I only mean to point out that depositionalization is a live alternative to equalization and I will not attempt any decisive claims about whether depositionalization or equalization is more just, all-things-considered. These judgements would require a complex array of empirical and normative considerations that will vary in different contexts. Instead of taking up these issues, my aim is to show (in the ‘Position-Defined Goods and Social Equality’ section) that there is a version of the convergence thesis that is not subject to them.

### Wealth as a Means to Self-Respect and Social Inclusion

What about the case of wealth as a means to self-respect and social inclusion? In this case it also seems that both the growth and depositionalization strategies are possible. On the one hand, we can ask whether growing the total amount of wealth in a society—making people better off in absolute terms, resource-wise—may improve the self-respect and social inclusion of the worse off, even despite inequality. On the other hand, we can ask whether it is possible to depositionalize wealth by weakening or removing the causal link between one’s relative level of wealth and its impact on self-respect and social inclusion.

One possibility suggested by Freiman involves appealing to what Rawls calls ‘non-comparing groups’, or what sociologists call ‘reference group’.^23^ These include clubs, sports teams, families—even one’s social class.^24^ These different groups provide a source of self-respect that is often somewhat insulated from other sources. For instance, a working-class person may gain a sense of self-respect through membership in their local sports club, and this might have a kind of eclipsing effect over dimensions in which they do not compare favourably, since people tend to emphasize those domains in which they do well.^25^

It may be the case, then, that self-respect can be promoted by fostering, proliferating, and insulating non-comparing groups. The more of these groups there are, and the more independent they are from one another, the more opportunity there will be to score well and gain self-respect from any one of them. This strategy of proliferating and strengthening non-comparing groups is served by material growth, insofar as these groups depend on material resources to develop and function: think, for example, of the creation of the Special Olympics and Paralympics, or the recent growth of E-sports (competitive video games).^26^ In this way, a wealthy but unequal society may provide more opportunities for self-respect than a poor but equal one. Interestingly, it seems here that the growth and depositionalization strategies overlap: by making many sources of self-respect salient, we weaken the contingent connection between relative wealth and self-respect.

We may doubt whether this suggestion is entirely compelling, however, insofar as people are likely to assign high importance to relative economic standing in societies as we know them. But even if we grant for the sake of argument that non-comparing groups function in this self-respect securing way, it is not obvious whether this strategy is plausible with regard to the concern with social inclusion. While non-comparing groups might provide a kind of social inclusion in relation to a particular group, this does not guarantee that a person will not suffer the kinds of loss of ‘associational goods’ and impairment of agency identified above.^27^ In other words, belonging to and ‘scoring well’ in one particular non-comparing group may not guarantee that one is regarded as ‘someone’ in society in general. For example, winning an event in the Paralympics may do much to bolster the self-respect of a person with a physical disability; but they may still suffer stigmatization in society more generally.

Perhaps, though, there are other ways in which material growth might support both self-respect and social inclusion; and perhaps there are other ways in which wealth can be depositionalized.^28^ For one, it seems plausible that self-respect and social inclusion of the worse off in the distribution of wealth is influenced not just by relative position, but by absolute level as well. Suppose there are two societies in which there is the same distance between the poorest and wealthiest groups, but in the first society the worse off have far fewer resources, in absolute terms, than in the other. In the second society the relatively poor group may be much less likely to suffer a loss of self-respect and social inclusion if they do well in absolute terms along key dimensions such as housing, education, healthcare, and so on. Self-respect and social inclusion of the worse off in the second society may be even more secure if the uses of wealth are limited. If having relatively more wealth only allows a person to buy more consumer goods, does not allow them to influence politics to a greater extent, or afford far superior healthcare or education, then having relatively less than others may be unthreatening to one’s self-respect and inclusion as a ‘full’ member of society.^29^ In this way, wealth might become depositionalized.

Again, we might worry that in societies as we know them, people do in fact assign high importance to relative difference in wealth. Thus, we might think that no matter how wealthy a society gets, if we take humans as they are, then great differences in wealth will more or less inevitably pose some threat to self-respect and social inclusion. Recall, for instance, the previous example of a super-wealthy society in which a cashmere shirt and crocodile leather shoes are necessary to count as ‘someone’. In such a society, limiting the uses of wealth would not be enough to depositionalize it. But this points to another possibility for depositionalization: that the *social meaning* of wealth may be subject to change. It may be possible, for instance, to adopt policies that encourage people to change the relevant kinds of attitudes. One example, Christian Schemmel suggests, could involve ‘pointing out in school that there is no good reason to link social acceptability to the wearing of brand name clothes’.^30^ In addition, policies might be adopted to encourage people to base their self-respect and attitudes towards others on the basis of their moral character or virtue.^31^ That these kinds of social change are live possibilities is evidenced by looking at different historical contexts. According to common standards in Maoist China, for example, great wealth marked one out for stigmatization, and poverty did not, instead serving as evidence of one’s commitment to the cause of the times.^32^

It is not obvious, of course, how feasible it is to change the relevant attitudes. As such, the convergence thesis might hold quite robustly, over an extensive range of cases. But what the previous considerations show is that it is also not obvious that equalizing wealth will always be the best or only way to benefit the worse off with regard to self-respect and social inclusion, and so the convergence thesis may fail to hold in a range of cases.

## Position-Defined Goods and Social Equality

To take stock so far, the convergence thesis holds that prioritarians, sufficientarians, and egalitarians all have reason to support the equal distribution of particular goods with positional aspects. This thesis has been advanced with regard to education and wealth, but it is not clear that removing or reducing inequalities of these goods will always be the only or best way to benefit the worse off or bring all up to a standard of sufficiency. In both cases, this can be explained in virtue of fact that the goods in question are ‘position-sensitive’. Position-sensitive goods possess two features, each of which corresponds to one of the strategies that might benefit the worse off instead of equal distribution. The first feature of position-sensitive goods is that they are a means to other goods which do not have a fixed supply. As we saw, relative position in the cases of education and wealth are means to employment or self-respect and social inclusion. But the overall supply of employment, as well as opportunities for self-respect and inclusion, can be grown in various ways, in some cases by permitting inequality. The second feature is that the value of position-sensitive goods to an agent only contingently depends on relative position. It follows from this feature that the situation of the worse off can be improved by weakening or eliminating this link. As a result of these two features, the convergence thesis is limited for these goods. In a range of cases, prioritarians or sufficientarians (and perhaps egalitarians too) may favour growth or depositionalization over equal (or more equal) distribution of these particular goods.

Does this mean the convergence thesis is unimportant? I do not think so. As noted, it may still hold in many cases. I have not meant to suggest that prioritarians or sufficientarians will never have reason to favour greater equality of education and wealth based on the above considerations. And in some cases, the convergence thesis may hold partially: priority or sufficiency may favour reducing inequality in combination with other strategies. What I want to stress, however, is that that favouring the equal distribution of these goods does not follow just in virtue of their position-sensitive features. It must also be shown that neither de-positionalization nor growth can do better. Whether growth, depositionalization, or equal distribution is best supported by a given principle of justice will involve complex and uncertain empirical considerations, such as how far inequalities really do create growth (perhaps a matter best left to economists) as well as how far groups really are ‘non-comparing’ (perhaps best left to sociologists). In addition, the issue will require further normative consideration (*e.g.,* which kinds of depositionalization would be favoured by justice) that I will not pursue here.

On the other hand, the convergence thesis would be of even greater interest if there are goods for which growth and depositionalization are not just infeasible, but impossible. I will show that this is the case for a different class of ‘position-defined’ goods. This result is significant because people’s civic status is both position-defined and an important (even fundamental) concern of justice, as argued for by social or ‘relational’ relational egalitarians. Moreover, a range of goods are ‘status-conferring’ in that they constitute a person’s civic status, so the version of the convergence thesis I propose bears on their distribution as well.

### Position-Defined Goods Characterized

What makes a good position-defined? We have seen that, for position-sensitive goods there is only a *contingent* connection between relative position and the good’s value to an agent, as in the cases of education-as-a-means-to-employment and wealth-as-a-means-to-self-respect and social inclusion. Position-defined goods, by contrast, are *defined by*, or *consist in* a particular relative position. Consider the good of ranking well in a competition; *e.g*., coming first place in a race. To enjoy this good *just is* to occupy a particular position relative to others—namely, performing better than them. While one might have an interest in performing well in absolute terms—by finishing the race in a certain amount of time, regardless of how others perform—this is not the same as the interest one might have in achieving a certain rank, say, taking home the gold medal.

This characteristic of position-defined goods bears on the applicability of the depositionalization and growth strategies. Notice, in the first case, that it would be simply confused to try to ‘depositionalize’ the value of coming first place in a race. Since the value of coming first consists in ranking above all others, an attempt to remove the positional aspect of this good would be to remove its value, or to replace it with something else. If gold medals in the Olympics were awarded according to some other criteria, say through lottery, or if every participant received one, they would no longer be valued in the same way, if at all. The second possibility, growing the supply of position-defined goods (or their uses), is also not entirely coherent. To be sure, it might seem like the supply of first place finishes is enlarged when people tie for first. Following this thought, it might be suggested we can grow the supply of first place finishes by awarding a gold medal to anyone who achieves a certain threshold of performance, in absolute terms. (This may also be regarded as an attempt at mitigating positionality.) But in the case of a tie for first place, or if ‘first-place’ were awarded on the basis of achieving a certain threshold, it would no longer have the same value, since it would no longer mark one out as the uniquely top performer (which is, presumably, what many competitors are after). In this way, depositionalizing or growing the supply by equalizing a position-defined good such as a gold medal involves a kind of incoherence, because it is defined in terms of ranking better than others.

In the case of position-defined goods, then, depositionalization and growth are not entirely coherent possibilities as they are for position-sensitive goods. This would be insignificant if all position-defined goods, such as rank in athletic competition, were not central to the theory of distributive justice. I argue, next, however, that there is another position-defined good—the rank or status people have as citizens—that *is* of central importance for distributive justice, as argued by social or relational approaches to egalitarianism.

### Civic-Status as a Position-Defined Good

To begin, I want to suggest how the notion of ‘status’ in general can be understood as a kind of institutional rank. First, consider how an ‘institution’ is defined by as Rawls as ‘a public system of rules, which defines offices and positions with their rights and duties, powers and immunities, and the like’.^33^ By defining various positions, institutions may assign their members various ranks, as ‘above’, ‘below’, or equal to others. In some cases, this ranking is explicit; but in other cases, it is less clearly so. Academic departments, for example, distinguish between various ranks of professors, with varied powers, responsibilities, and benefits. Martial arts clubs distinguish between the ‘Sensei’ and the various ranks of students (black belts are above the orange belts who are above the yellow belts, and so on). Families, less explicitly, may rank parents as the ‘head(s) of the household’ with older children being ‘above’ the younger ones, having greater rights and responsibilities.^34^ Status, in this sense of an institutional rank, is a position-defined good. What it is to hold a particular status is defined by a particular relative position—as above, below, or equal to others.^35^

Of particular importance for social justice is civic status—the rank people have as citizens under social, political, and legal institutions.^36^ We can think of civic status as involving three elements: one *objective;* another, *communicative;* and a third, *intersubjective*, outlined as follows.^37^

*Objective*: This element specifies the distribution of goods that are required to confer people a status as equal citizens. This may involve, for example, equal basic liberties; constraints on inequalities of income and wealth, and adequate education and healthcare.^38^

*Communicative*: It is not enough that people are given these status-conferring goods on any basis (*e.g.*, their being equally deserving, intelligent, and so on). Instead, these goods are given to people on the basis that they are antecedently equals; and their distribution publicly communicates respect for persons as equals.^39^

*Intersubjective*: The equal distribution of status-conferring goods is a matter of common knowledge, setting a public standard for how people ought to regard and treat each other as equal citizens.

I use the term civic status to register that equal status in this sense is compatible with inequalities of social esteem or what might be meant by ‘social status’. Some may enjoy a high level of social status or esteem in virtue of their celebrity as actors, performance as athletes, or their possession of rare paintings. But this sort of local, domain-specific ‘social status’ inequality is compatible with all sharing a kind of global and domain-generic status as equal citizens. Someone being highly esteemed in a domain such as musical performance is no reason for them to be regarded as belonging to a fundamentally different class of citizens, provided all share the same basic liberties, if economic inequalities are not too large, and so on, however the *objective* element of the account is determined.^40^

The intersubjective and communicative aspects are important because they are required for people to enjoy the position-defined good of equal civic status, as opposed to ‘merely’ enjoying a particular set of goods. To see this, imagine two cases in which each person in a society has an equal amount of the kinds of status-conferring goods identified above. In one society, this is a matter of common knowledge: each person knows that they have the same set of status-conferring goods as anyone else, they know that others know this, they know others know this, and so on (and so the intersubjective dimension is satisfied).^41^ Moreover, there is common knowledge that each person is entitled to these goods on the basis that they *are equals* (and so the communicative dimension is satisfied).^42^ In the second case, by contrast, each person does in fact objectively possess an equal set of status-conferring goods, but they are unaware of what others have, and the equal distribution does not publicly communicate any particular message. It is only in the first case that each person enjoys the position-defined good of having a status as an equal. Because the intersubjective and communicative dimensions are satisfied, there is a public ranking of people as equal citizens which is not present in the second case. In this sense, then, civic status is a good that is defined by a relative position of equality and involves objective, intersubjective, and communicative aspects.

To be sure, there seems to be a sense in which a person’s civic status may not be entirely determined by relative position. We can imagine two societies in which all enjoy a status as equals, but in one society the level of status-conferring goods—*e.g.*, rights and liberties, income and wealth—is much higher or more extensive than the other. Here it seems we would want to say that the status people have as equals is ‘higher’ in the society in which the enjoyment of status-conferring goods is greater. In this case, however, what is greater is the enjoyment of non-positional goods. The position-defined good of civic status they confer is the same in each society. By analogy, all runners in a race may simultaneously achieve better scores while their relative ranks stay fixed.

### The Convergence Thesis Revisited

Since civic status is a position-defined good, depositionalization and growth are not viable strategies to benefit the worse off. First, it is not possible to reduce the extent to which the value of civic status depends on relative position, since it is defined by or consists in relative position. What matters is not that people have a particular degree of civic status independently from what others have; what matters is that they have a civic status as an equal.^43^ Second, the growth strategy relies on the possibility that the worse off might enjoy a greater amount of a good under an unequal distribution than they would under equality. In other words, benefitting the worse off favours an unequally divided pie, if its smaller slices are bigger than a smaller pie equally shared. As matter of definition, however, some holding a high civic status entails that others hold a low status. It follows, then, that an equal distribution of civic status is better for the worse off than any other distribution (all else equal).

For this reason, there would seem to be little reason to adopt any principle other than equality insofar as we are concerned with benefitting the worse off with regard to this good. Consider, for example Rawls’s difference principle, which permits inequality when it provides the worse off more benefits than they would enjoy under equality. This principle applied to civic status would be equivalent to equality. It would also seem to make little sense to adopt a principle of civic status priority or sufficiency over equality. Perhaps a prioritarian may permit a class of inferiors if they do not assign them a very high degree of priority, or if they judged there to be enough aggregate benefit from allowing a class of superiors. While divergence is still possible in this sense, this kind of view is not likely to be found very plausible, insofar as being ranked as an inferior or second-class citizen has very bad effects on people’s wellbeing.

More plausible, perhaps, is the case of ‘relational sufficiency’, which requires that people are able to ‘relate as sufficients’ rather than as equals.^44^ Kasper Lippert-Rasmussen suggests, for instance, that this view might permit ‘unequal relations between a worshipping religious follower and a sufficiently respectful guru…or a boss with suitably circumscribed powers and an employee with alternative employment opportunities’.^45^ Similarly, John Tosi claims that people do not need equal status ‘across the board’: ‘[t]hey do not need a workplace without hierarchical management, for instance, or the elimination of other voluntary associations that admit of distinctions of status.’^46^

While these suggestions have some plausibility, they do not seem to involve inequality of civic status. Indeed, if we share the intuition that the kind of unequal relations just mentioned are unobjectionable, it seems to me that we do so *because* we are assuming a background of equal civic status. As noted before, equality of civic status (and, I think, social or relational equality more broadly) need not involve everyone having exactly the same status in all social dimensions. As Samuel Scheffler noted in an early discussion ‘differences of rank, power, and status are endemic to human social life…’ but further, he suggests it does not seem necessary, ‘in order for a relationship to qualify as having an egalitarian character, that it should be altogether unmarked by distinctions of rank or status’.^47^

Developing this thought, social egalitarians can identify a particularly salient dimension such that if people are equal in that dimension, distinctions of rank and status in other domains may not damage the overall egalitarian character of the relationship. On my account, equal citizenship is this particularly salient dimension. If people rank as equal citizens, then some forms of hierarchy in the workplace, or religious organizations, and so on, may not upset this fundamental equal relationship, especially if the kinds of hierarchy in these contexts are limited in particular ways. It is telling, in this regard, that the examples of relational sufficiency above involve, for instance, a boss with ‘circumscribed powers’ and an employee with ‘alternative opportunities’, or *voluntary* associations that admit of distinctions of status. None of these examples of supposed relational inequality involve people relating as unequal citizens; and if they did, then we would have reason to find them objectionable. Thus, with regard to civic status, sufficiency is not a plausible alternative to equality. Put another way, what it is to have enough civic status is for it to be equal.

On this basis, there is a compelling case for the convergence thesis when applied to the good of civic status. This is significant, because there are further implications for the range of status-conferring goods (whatever they are determined to be exactly), such as the basic liberties, income and wealth, education, healthcare, and so on. Since these goods function to rank people as citizens, and so have distinctive positional aspects in this regard, this impacts what is required to benefit the worse off, or give all ‘enough’ of these goods. While it would be beyond the scope of this paper to give a complete account of these implications, I will try to further illustrate the idea that there is distinctive concern at stake.

In the case of the basic liberties, for example, it would appear that anything other than an equal distribution would function to rank some as inferiors, and so be unacceptable.^48^ For other status-conferring goods, however, strict equality is not likely to be required. Nevertheless, a greater degree of equality may be required on the basis of a concern with realizing equal civic status, than if we are concerned with other positional aspects—for example, labour market opportunity.

Consider Debra Satz’s account of ‘adequacy’, or sufficiency in the distribution of education, where this threshold is specified by what a person needs to possess a status as an equal citizen.^49^ She claims her conception of sufficiency converges with egalitarian considerations, because ‘adequacy is not only a function of the bottom of the distribution but also of the top of the distribution’.^50^ Furthermore, this conception of sufficiency may support removing advantages from the top, without transferring them, since ‘citizens are not equals when there is a closed intergenerational social elite with disproportionate access to society’s positions of political and economic power’.^51^

These claims are substantiated by the position-defined nature of civic status. Whether one has an adequate level of education to possess the status of an equal citizen depends on how much they have compared to others, and whether the overall distribution of education is equal enough. By contrast, consider education-as-a-means-to-employment. Whether one has ‘enough’ of this good appears, in principle, to be compatible with any range of inequality. For example, on sufficientarian (or prioritarian) grounds, the existence of a social elite may not be unjust if it were to improve the job prospects of the worse off from what they would enjoy under greater equality. This would be a case in which the convergence thesis no longer holds. Sufficiency or priority of education-as-a-means-to-employment would not require greater equality; and it would not be an improvement of justice to remove advantages from the top. On the other hand, the convergence thesis would still hold in this case for sufficiency of education-for-equal-civic-status. While the existence of a social elite may not necessarily threaten the job prospects of the worse off, it would threaten their equal civic status. In this way, the convergence thesis holds more robustly (over a more extensive range of possibilities) for education-for-equal-civic status than for education-as-a-means-to-employment.

It is worth pointing out, however, that this claim depends on the assumption that greater education can make a person a ‘social elite’, defined above  in terms of having ‘disproportionate access to society’s positions of political and economic power’. One could imagine, alternatively, a society that has substantial inequality of education, but where having greater education provided no greater power over others. We could imagine, too, that education is not particularly valued, such that people with more of it are not thought of as ‘better-than’ in any significant way. In this case, inequality of education would not appear to undermine equality of civic status. This suggests two points (familiar from the discussion of wealth earlier in the paper): first, that whether inequality of a status-conferring good is incompatible with social equality depends on the permitted uses of that good; and second, it depends on the social meaning of the good—that is, whether possession of it is something that is valued or regarded in the relevant ways by the relevant community. Nevertheless, given that education is likely to provide a range of advantages in most societies, and to be regarded as something important, concern with equality of civic status is likely to require significant limitations on inequalities of education.

The kind of argument just suggested could be generalized to other status-conferring goods. For example, Brighouse and Swift note that healthcare is ‘latently’ positional, because being healthier than others provides an advantage on the job market.^52^ On the approach taken here, we can point out a further positional aspect of healthcare: inequalities of healthcare may preclude some from being civic equals. Interestingly, because of the central importance of health for all aspects of life, it does not seem that inequality of healthcare may be rendered more compatible with social equality by limiting its uses, as in the case of education; and it also seems that the social meaning of health is likely to remain fairly stable across different contexts. Thus, a concern with equality of civic status is also likely to support significant limitations on inequalities of healthcare.^53^

## Conclusion

The category of positional goods warrants examination by political philosophers because they function differently than other goods in the theory of justice. The aim of this paper has been to advance this project. I explored the convergence thesis, or the claim that there may be less at stake than commonly thought between seemingly divergent principles of justice—priority, sufficiency, equality—with regard to positional goods. This idea has been notably advanced in the cases of education-as-a-means-to-employment and wealth-as-a-means-to-self-respect and social inclusion. It may not work so well in those cases. But it works perfectly in a case not considered: that of civic status, the good of central concern on social or ‘relational’ accounts of equality. The difference hinges on a distinction between position-sensitive and position-defined goods. This account is significant, I suggested, because a range of goods, such as education and healthcare, have distinctive positional aspects in virtue of their status-conferring role, and examining these aspects in further detail may be a fruitful topic of further research.

## Footnotes

1 For the original discussion of positional goods see (Hirsch 1995); for discussions in the philosophical literature see (Goodin 1995), (Scanlon 2003), (Brighouse and Swift 2006), (Satz 2007), (Ypi 2012), (Freiman 2014), (Halliday 2015), (Axelsen and Nielsen 2015), (Nielsen and Axelsen 2017), and (Harel Ben-Shahar 2018).

2 Some critics doubt a concern for equal distribution per se is important, involving irrationality, or worse, an inappropriate kind of envy towards the better off. Why should we support equal distribution if the worse off could fare better in an unequal distribution than they would under equality? Supporting equal distribution seems to involve holding that there is at least something valuable in ‘levelling down’: removing benefits from some while benefitting no one. For these reasons, many philosophers reject egalitarianism in favour of other principles such as giving each enough (sufficiency) or maximizing with greater weight given to the worse off (priority). See (Parfit 2002) and (Frankfurt 1987).

3 See (Brighouse and Swift 2006), (Scanlon 2003), (Goodin 1995), (Satz 2007), (Ypi 2012), (Axelsen and Nielsen 2015), and (Nielsen and Axelsen 2017). Some writers such as Brighouse and Swift focus primarily on the case of priortarianism, while others, such as Axelsen and Nielsen, as well as Satz, focus on sufficiency.

4 Brighouse and Swift (2006).

5 I borrow this term (and similar to follow) from Freiman (2014).

6 Adam Smith notes the context-sensitivity of the point, as follows: ‘The Greeks and Romans lived, I suppose, very comfortably though they had no linen. But in the present times, through the greater part of Europe, a creditable day-labourer would be ashamed to appear in public without a linen shirt, the want of which would be supposed to denote that disgraceful degree of poverty which, it is presumed, nobody can well fall into without extreme bad conduct. Custom, in the same manner, has rendered leather shoes a necessary of life in England. The poorest creditable person of either sex would be ashamed to appear in public without them’. (Smith 1993), bk. 5, Ch. 2.

7 Rawls suggests that feelings of envy under such circumstances are appropriate or reasonable: ‘sometimes the circumstances evoking envy are so compelling that given human beings as they are, no one can reasonably be asked to overcome his rancorous feelings. A person’s lesser position … may be so great as to wound his self-respect’. (Rawls 1999), p. 535.

8 Rawls (1999), p. 131. There are various kinds of attitudes at stake here that are sometimes distinguished in the literature. Roughly, one notion of self-respect involves regarding oneself as owed certain forms of moral treatment; the other notion (sometimes called self-esteem) involves regarding oneself as scoring well according to some standard (*e.g.,* morality, athletics, music, and so on). These notions are defined with various terms such as ‘recognition vs. appraisal’; ‘entitlement vs. standard’; and ‘standing vs. standard’ self-respect. See, respectively, (Darwall 1977); (Bird 2010); (Schemmel 2021), Ch. 6.

9 Brighouse and Swift (2006), p. 481.

10 Scanlon (2018), p. 26.

11 *Ibid.,* p. 30.

12 Consider the example, discussed by Scanlon, of an African American woman whose designer clothing apparently enabled her to have welfare payments for the granddaughter of her neighbour restored (while her neighbour had been turned away). See (Scanlon 2018), p. 30, discussing an online editorial (Cottom 2013).

13 The concern in the cases discussed is that inequalities are not too large (intuitively, some inequality with regard to education and wealth is unobjectionable). However, in the case of other positional goods—votes, for example—there appears to be reason to be concerned with any form of inequality. On the importance for the size of inequalities and positional goods, see (Harel Ben Shahar 2018).

14 (Scanlon 2003), p. 204.

15 Freiman (2014).

16 Notice that the suggestion here is not to grow the supply of education itself (*e.g*., creating more schools). This would not likely help the problem, because insofar as there remain inequalities of education, the job prospects of the relatively less educated will suffer.

17 For similar reasons, Rawls prefers the difference principle over a strictly equal distribution of income and wealth (Rawls 1999).

18 Freiman (2014).

19 Brighouse and Swift (2006), p. 488.

20 Goodin (1995), p. 257. Harel Ben Shahar (2018), p. 111–113.

21 Satz (2007), p. 644.

22 As noted previously, it is also possible that egalitarians might favour these strategies as well, if they bring about an equal distribution of wellbeing, or whatever metric of justice is ultimately taken to matter.

23 (Freiman 2014).

24 ‘The plurality of associations in a well-ordered society, each with its own secure internal life, tends to reduce the visibility, or at least the painful visibility, of variations in men’s prospects. For we tend to compare our circumstances with others in the same or in a similar group as ourselves, or in positions that we regard as relevant to our aspirations. The various associations in society tend to divide it into so many noncomparing groups, the discrepancies between these divisions not attracting the kind of attention which unsettles the lives of those less well placed’. (Rawls 1999), pp. 536–537.

25 Here is Nozick on the issue: ‘Everyone might view themselves as at the upper end of a distribution through the perspective of the particular weights he assigns. The fewer the dimensions, the less the opportunity for an individual successfully to use as a basis for self esteem a nonuniform weighting strategy that gives greater weight to the dimension he scores highly in’. (Nozick 2013), p. 245.

26 Freiman (2014), p. 349. Cf. Anderson (2012).

27 Freiman seems to hold that the non-comparing group-specific kind of inclusion is all that exists, suggesting ‘there is no monolithic, homogenous good that constitutes social inclusion in a pluralistic society’ (Freiman 2014), p. 352. This suggestion, however, ignores the sense in which people can suffer exclusion in the general ways identified in this paper.

28 This idea seems to be at work in Adam Smith’s discussion of the commercialization that took place early in the Industrial Revolution. Smith saw commercialization as bringing growth and efficiency as benefits; but also important, he thought, was that it opened the possibility for the rich to convert their wealth into something other than power. The rich, he says, ‘gave up their power in the wantonness of plenty, for trinkets and baubles, fitter to be the playthings of children than the serious pursuits of men, they became as insignificant as any substantial burgher or tradesman in a city’ (Smith 1993), bk. 3, Ch. 4. On Smith’s account, then, commercialization created growth; but at the same time, there was a depositionalizing effect. The wealthy no longer used their wealth to exercise power over others, but instead sought material vanities, which could be regarded as ‘childish’ by others.

29 This is the strategy of ‘blocked exchanges’ identified by (Walzer 2008). See also (Miller 1995). Cf. (Schemmel 2021), pp. 249–250.

30 Schemmel (2021), p. 250.

31 I would like to thank an anonymous referee for this suggestion.

32 See discussion in (Ci 2013) and (Scanlon 2018), Ch. 3.

33 Rawls (1999), pp. 47–48.

34 An institutional ranking may be even less explicit, yet still present: in the global order, for example, the ‘Global North’ ranks ‘above’ the ‘Global South’, and certain passports are ‘stronger’ than others, allowing their possessors to travel more freely, and so on.

35 Rawls notes this point as follows: ‘Status is a positional good…in a status system, not everyone can have the highest rank. High status assumes other positions beneath it; so if we seek a higher status for ourselves, we in effect support a scheme that entails others having lower status’ (Rawls 2001), p. 132.

36 Social equality can be understood to be important on several different grounds, including grounds of personal and impersonal value. See (Tomlin 2014). Most significantly, however, and perhaps least controversially, I hold that a concern with equality of civic status is grounded in the requirement that the state show respect for its members as equals—what Dworkin calls the ‘sovereign virtue’ or what Kymlicka calls the ‘egalitarian plateau’. See (Dworkin 2002), (Kymlicka 2002). No state respects its members as equals if it assigns some a status as second class or inferior citizens.

37 It is worth nothing that this understanding of social equality characterized in terms of an institutional rank differs from several recent accounts that characterize social equality in terms of particular interpersonal relations and dispositions. See (Scheffler 2015) (Kolodny 2014). See also (Viehoff 2019), for a useful characterization of political and interpersonal conceptions of social equality. The institutional approach favoured here is closer to the views developed by Elizabeth Anderson and Christian Schemmel. See (Anderson 1999) and (Schemmel 2021).

38 It is a difficult matter to resolve the question of exactly what distribution of goods is required to confer the right kind of equal status. For example, we could consider Rawls’s two principles as one possible way to spell out in detail the requirements of equality of civic status. And there are other possibilities, such as Elizabeth Anderson’s ‘democratic equality’ or Philip Pettit’s republicanism. For the most recent and developed account, see (Schemmel 2021).

39 On the communicative or expressive dimension, see (Anderson and Pildes 2000; Shiffrin 2017; Voigt 2018; Schemmel 2021).

40 This is not to say that it is not possible for some norms of esteem to undermine equality of civic status—for instance, if members of some groups are systematically assigned lesser esteem for comparable performances in a particular domain. See (Schemmel 2021), Ch. 6.

41 Lewis (1969).

42 We could imagine a society in which each person is given an equal set of status-conferring goods, but not on the basis of people’s equality. Suppose for instance, that one group is regarded as inferior, but given an equal set of status-conferring goods on the basis of pity, or the requirements of ‘noblesse oblige’. Intuitively, this would not suffice to give people a status as equal citizens.

43 Cf. Gheaus (2018), pp. 61–62.

44 Lippert-Rasmussen (2018), pp. 9–11; Tosi (2019).

45 Lippert-Rasmussen (2018), p. 10.

46 Tosi (2019), p. 55.

47 Scheffler (2005), pp. 17–18.

48 Cf. Rawls (1999), p. 477. See also Cass (2021).

49 Satz (2007). See also Anderson (2007).

50 Satz (2007), p. 638.

51 *Ibid.*

52 Brighouse and Swift (2006).

53 See Schemmel (2021), Ch. 9, for a relational egalitarian account of healthcare.
